# *CALCRL* Gene is a Suitable Prognostic Factor in AML/ETO^+^ AML Patients

**DOI:** 10.1155/2022/3024360

**Published:** 2022-03-16

**Authors:** Rongrong Wang, Miao Li, Yujia Bai, Yang Jiao, Xiaofei Qi

**Affiliations:** ^1^Department of Hematology, The First Affiliated Hospital of Soochow University, Suzhou, China; ^2^National Clinical Research Center for Hematologic Diseases, Jiangsu Institute of Hematology, Collaborative Innovation Center of Hematology, Suzhou, China; ^3^Institute of Blood and Marrow Transplantation, Soochow University, Suzhou, China; ^4^Cyrus Tang Hematology Center, Soochow University, Suzhou, China; ^5^State Key Laboratory of Radiation Medicine and Protection, School of Radiation Medicine and Protection, Soochow University, Suzhou, China; ^6^Department of Urology, The First Affiliated Hospital of Soochow University, Suzhou, China

## Abstract

**Introduction:**

The t(8 ; 21) translocation is the most common chromosomal abnormality in human acute myeloid leukemia (AML) subtype 2 (M2), which forms the AML/ETO fusion gene. However, AML/ETO alone does not necessarily cause leukemia. Other factors are thought to contribute to the disease. Calcitonin receptor-like (CALCRL), a G-protein-coupled neuropeptide receptor, is involved in various biological processes, such as colony formation and drug resistance.

**Methods:**

First, The Cancer Genome Atlas (TCGA) and Gene Expression Omnibus (GEO) databases were used to determine any differences in *CALCRL* expression in AML patients with and without AML/ETO and the prognostic significance of *CALCRL* expression in AML patients was further evaluated. Next, we detected the *CALCRL* expression level in 67 AML/ETO^+^ AML patients and 16 patients with nonmalignant hematological diseases using qRT-PCR and identified its prognostic relevance.

**Results:**

Individuals in the group expressing low levels of *CALCRL* had a longer median survival time. In AML/ETO^+^ AML patients, higher mRNA levels of *CALCRL* were observed before treatment, which decreased after the complete remission that followed multiple chemotherapy sessions. Clinical features indicated that more patients in the CALCRL^high^ group also had c-kit mutations compared with patients in other groups. Overall survival (OS) was longer in patients with lower levels of *CALCRL* expression, especially in patients with c-kit mutations or with more blast cells in bone marrow (BM). In addition, a longer OS was observed in the CALCRL^low^ group after hematopoietic stem cell transplantation (HSCT).

**Conclusions:**

This preliminary study indicates that CALCRL could serve as a suitable prognostic factor in AML/ETO^+^ AML patients.

## 1. Introduction

The AML/ETO fusion transcription factor is one of the most common chromosomal abnormalities detected in human acute myeloid leukemia (AML) subtype 2 (M2). However, AML/ETO alone does not necessarily cause leukemia [1–3]. Moreover, AML/ETO^+^ AML is currently believed to have a good prognosis. However, due to the leukemic clonal diversity, the recurrence rate is high. All these implied that additional factors must participate in the development of AML in those patients [4, 5].

Our previous studies showed that H22954, a novel long noncoding RNA, may play an important role in the pathogenesis of AML [6]. To understand the mechanism underlying the antitumor activity of H22954, we conducted a microarray study to examine differentially expressed genes in stable K562 cells that did and did not express H22954. Calcitonin receptor like (*CALCRL*) was one of several genes whose expression decreased when H22954 was highly expressed. CALCRL is a G protein-coupled receptor engaged in regulating the concentration of calcium ions in cells. It inhibits cell proliferation and angiogenesis [7]. CALCRL also contributes to the drug resistance in AML by controlling the ADM-CALCRL axis [8, 9]. However, the relationship between *CALCRL* expression and the clinical features and prognosis of AML patients have only rarely been addressed. We conducted the present study to address these issues. We used The Cancer Genome Atlas (TCGA) and Gene Expression Omnibus (GEO) datasets and also enrolled 67 newly diagnosed AML/ETO^+^ AML patients in this study. We found that the mRNA expression level of *CALCRL* is correlated with the diagnosis, survival, and prognosis of AML/ETO^+^ AML patients. This may provide new ideas for the diagnosis and treatment of AML.

## 2. Materials and Methods

### 2.1. TCGA and GEO Datasets

Clinical information of AML patients was obtained from TCGA dataset (https://portal.gdc.cancer.gov/) following the guidelines and policies of our hospital. Kaplan–Meier (KM) survival analysis with the log-rank test was also used to compare the survival between the AML patients with and without AML/ETO in the GEO dataset (GSE61804). For KM curves, *P* values and hazard ratios (HRs) with a 95% confidence interval (CI) were generated by log-rank tests.

### 2.2. Study Cohort

In total, 67 newly diagnosed AML/ETO^+^ AML patients were recruited. They had been diagnosed based on morphology, immunology, cytogenetics, and molecular biology (MICM) [6, 10] at The First Affiliated Hospital of Soochow University between January 2015 and December 2018. As a control group, 16 patients with nonmalignant hematologic disease were enrolled. The study protocol was approved by the hospital's institutional ethical committee, and informed written consent was obtained from all of the patients.

### 2.3. Detection of CALCRL Expression by Quantitative RT-PCR (qRT-PCR)

Bone marrow mononuclear cells (MNCs) were collected from the experimental group and the control group. Total RNA (500 ng) was directly reverse-transcribed into cDNA. PCR primers and TaqMan probes were designed using Primer Express 2.0 software with published sequence data from the NCBI database. Amplification reactions contained 2 *μ*l cDNA, 1 *μ*l 20x buffer, 2 *μ*l MgCl_2_, 4 *μ*l 5x Q-Solution, 0.8 mM dNTPs, 2 *μ*M primers, 1 *μ*M probes, and 1 U HotStar (QIAGEN, Germany) [11]. *ABL* served as the internal control, and the relative expression level of *CALCRL* was calculated using the gene relative quantitative method. The primers for *CALCRL* were CALCRL-F, 5′-GCAGCAGCTACCTAGCTTGAA-3′; CALCRL-R, 5′-TTCACGCCTTCTTCCGACTC-3′; and CALCRL-probe, 5′-FAM-ACTGCAGTGGCCAACAACCAGGCCT-TAMRA-3′. The primers for *ABL* were ABL-F, 5′-CGCTGACCATCAATAAGGA-3′; ABL-R, 5′-CACTCAGACCCTGAGGCTCAA-3′; and ABL-probe, 5′-FAM-TGCTTCTGATGGCAAGCTCTACGTCTCCT-TAMRA-3′.

### 2.4. Treatment

The induction chemotherapy regimen used to treat the patients was as follows: IA (IDA + Ara-C) and DA (DNR + Ara-C). Whenever there was no remission after induction chemotherapy, the induction chemotherapy regimen was changed. Postremission treatment included consolidation chemotherapy and hematopoietic stem cell transplantation. In addition to this induction regimen, the consolidation chemotherapy regimen also included medium- and high-dose cytarabine (HD-Ara-C/MD-Ara-C), single-drug chemotherapy, and other treatments. All of the patients were followed up until December 2018 or death via consultation of hospitalization medical records, telephone calls, and other methods.

### 2.5. Statistical Analysis

The data were analyzed using SPSS20. The mean values of the two groups were compared by *t*-test, and the mean values of multiple samples were analyzed by variance analysis. The survival curve was drawn using GraphPad Prism 8.2.1 software. Survival probabilities were estimated using the KM method and the log-rank test. *P* value < 0.05 was considered to be indicative of statistical significance.

## 3. Results

### 3.1. CALCRL Expression in AML Patients and Controls

To understand the mechanism underlying the antitumor activity of H22954, we compared gene expression profiles in the stable K562 cells that did and did not overexpress H22954 through microarray analysis and found 1613 differentially expressed genes that had an expression fold change of <0.5 and *P* < 0.05 in H22954-overexpressing cells (Supplementary Fig. S1). In parallel, BLAST analysis showed 400 human genes to be potential H22954 target genes in the TargetScanHuman 6.0 database. In total, 81 genes were present in both of these sets. Next, we performed the prognostic analysis of these 81 genes in the TCGA datasets. The *CALCRL* gene showed the strongest relationship to overall survival (OS) in AML.

**Figure 1:**
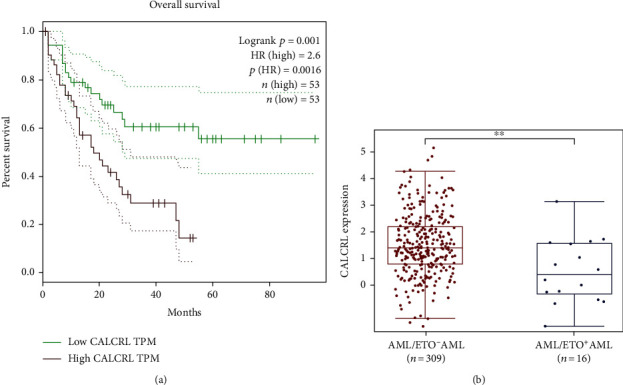
(a) *CALCRL* gene expression and survival time and survival status in the TCGA dataset. (b) *CALCRL* expression in AML with AML/ETO (*n* = 16) or without AML/ETO (*n* = 309).

The full TCGA cohort contained 106 AML samples. All of the samples were split into high- and low-expression groups based upon median *CALCRL* gene expression. As shown in Figure 1(a), the median survival time was about 20 months in the high-expression group vs. >80 months in the low-expression group. An increase in *CALCRL* expression was associated with inferior OS (HR, 2.6; 95% CI, 1.473–4.591; *P* < 0.05) (Figure 1(a)). In the GEO database GSE61804, there were 16 AML/ETO^+^ AML patients and 309 AML/ETO^−^ AML patients. Lower *CALCRL* mRNA levels were observed in the presence than in the absence of AML/ETO (Figure 1(b)).

**Figure 2:**
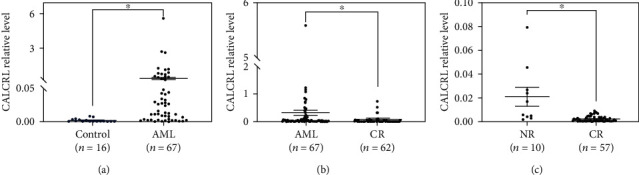
(a) Quantification of *CALCRL* mRNAs in the BMNC bone marrow samples from patients with AML/ETO^+^ AML (*n* = 67) or nonmalignant blood disease (*n* = 16). (b) Correlation between *CALCRL* expression and clinical phase. *CALCRL* mRNAs were detected in pretreated patients (*n* = 67) and remission phase patients (*n* = 62). cDNAs from all samples were subjected to real-time quantitative RT-PCR analysis with primers specific for *CALCRL* and *ABL*. The ratio of the abundance of *CALCRL* transcripts to that of *ABL* transcripts (*CALCRL/ABL*) was calculated for statistical analysis. (c) Correlation between *CALCRL* expression before treatment and remission rate after the first course of induction chemotherapy.

To determine the relationship between *CALCRL* expression levels and the clinical features or prognosis of AML patients, 67 primary AML/ETO^+^ AML patients were enrolled, including 40 men and 27 women, with a median age of 35 (range, 15–57) years. Additionally, 16 patients (8 men and 8 women) with nonmalignant hematologic disease (iron-deficiency anemia or megaloblastic anemia) were set as the control group, who had a median age of 31 (range, 22–51) years. *CALCRL* mRNA expression was significantly higher in the 67 patients with primary AML (AML/ETO^+^) than in the nonmalignant hematologic group (*P* < 0.05) (Figure 2(a)).

After multiple chemotherapy courses, 62 of 67 patients achieved clinical remission (CR). *CALCRL* expression was lower in CR patients (*P* < 0.05) (Figure 2(b)). After the first course of chemotherapy, 57 cases achieved CR and 10 cases did not reach remission (NR). Before treatment, lower *CALCRL* mRNA levels were observed in bone marrow mononuclear cells (MNCs) in the CR group than in the NR group (*P* < 0.05) (Figure 2(c)).

The cohort of all 67 AML/ETO^+^AML patients was divided into two expression groups based on the median value: 34 patients were included in the CALCRL^low^ group, and 33 patients were included in the CALCRL^high^ group. Basic clinical features were similar between the two groups, including white blood cell counts, neutrophils, hemoglobin, platelets, and the percentage of blasts in the bone marrow. No significant correlations were detected between the mRNA expression of *CALCRL* and the risk stratification of leukemia (Table 1). The proportions of *CEBPEA* and *FLT3* mutations detected were similar in both groups. Although we do not know why, there were more patients with c-kit mutations (25/29) in the CALCRL^high^ group than in the CALCRL^low^ group (14/27) (*P* < 0.05) (Table 2).

### 3.2. Relationship between CALCRL mRNA Expression and Survival in AML/ETO^+^ AML Patients

**Figure 3:**
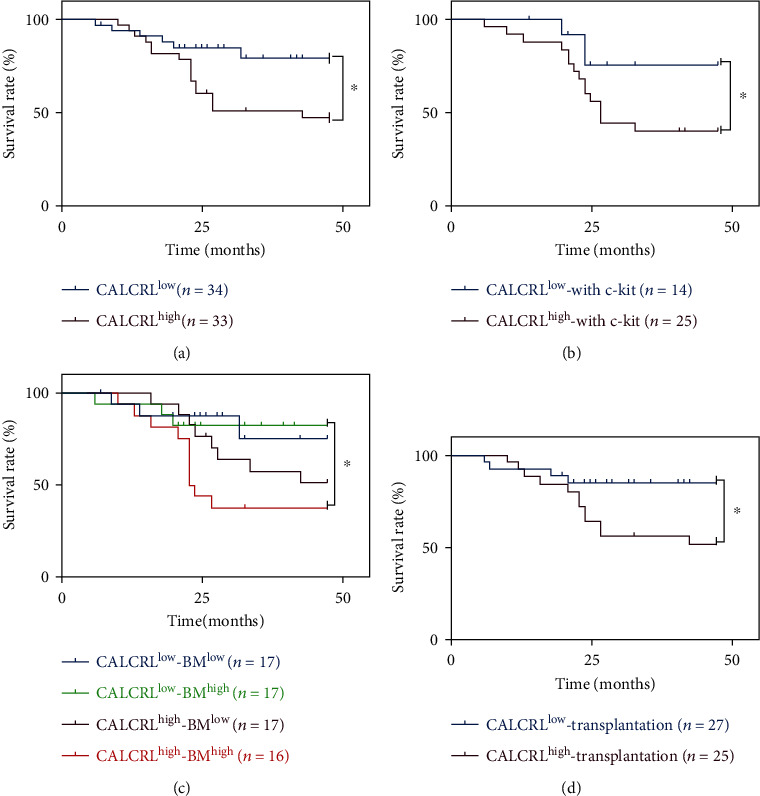
(a) Correlation between *CALCRL* expression and survival outcomes. (b) Correlation between *CALCRL* expressions combined with bone marrow blast cells and survival outcomes. (c) Correlation between *CALCRL* expressions combined with c-kit mutations and survival outcomes. (d) Overall survival (OS) rates in AML/ETO^+^ AML patients after HSCT with *CALCRL* expression levels high (green) (*n* = 25) or low (blue) (*n* = 27) the median value. Statistical analysis was based on survival analysis.

The survival analysis indicated a significantly negative correlation between the expression of *CALCRL* and OS (*P* < 0.05) in AML/ETO^+^ AML patients (Figure 3(a)). With concomitant c-kit mutations, *CALCRL* mRNA expression levels and the patient's OS were also negatively correlated (*P* < 0.05) (Figure 3(b)). CALCRL has been reported to be related to leukemic cells, so the patients were divided into two groups for further analysis based on the median proportion (46%) of bone marrow blast cells: a group with many blast cells in the bone marrow (BM^high^) and a BM^low^ group. In the BM^low^ patients, the survival time was similar regardless of the level of *CALCRL* mRNA expression. However, in the BM^high^ group, the OS was shorter in those with a higher level of *CALCRL* mRNA expression (*P* < 0.05) (Figure 3(c)).

Of 67 patients, 52 patients received hematopoietic stem cell transplantation (HSCT). Of these, 27 patients belonged to the CALCRL^low^ group and 25 patients were in the CALCRL^high^ group. The OS of patients in the CALCRL^low^ group was significantly longer than that in the CALCRL^high^ group (*P* < 0.05) following HSCT (Figure 3(d)).

## 4. Discussion

AML is a malignant hematological disease that is affected by genetic and epigenetic factors [6]. Various molecules and genetic alterations have been identified as contributing factors in AML. Our previous work indicated that H22954 plays a critical role in AML; 81 genes were found to be potential H22954 target genes in a microarray study and bioinformatics analysis. Of these, *CALCRL* was shown to be closely related to the OS in AML.

CALCRL is a G protein-coupled neuropeptide receptor involved in blood pressure regulation, cell proliferation, apoptosis regulation, vascular biology, and inflammation [12–15]. In solid tumors, antibody-mediated inhibition of CALCRL signaling has been shown to reduce tumor growth [16–18] and it is currently being examined as a new and promising candidate therapeutic target for AML [8, 9].

To determine the relationship between CALCRL expression and the clinical features and prognosis of AML, AML/ETO^+^ AML patients were enrolled in the present study, taking the advance of the AML1/ETO fusion gene as the biomarker of AML in these patients. It was deemed unethical to aspirate bone marrow from healthy controls, so nonmalignant hematologic patients were chosen as controls. Our investigation showed that there were higher mRNA levels of *CALCRL* in pretreated AML/ETO^+^ AML patients than in control samples and these levels decreased when patients got CR. More AML/ETO^+^ AML patients with high levels of *CALCRL* expression also had c-kit mutations than patients in other groups.

Survival and prognostic analysis showed that patients with less *CALCRL* expression more readily achieved CR after the first course of chemotherapy and there was a negative correlation between the mRNA expression of *CALCRL* and patient survival time. This correlation was especially strong in BM^high^ patients. Patients with lower *CALCRL* mRNA expression may reap greater benefits from HSCT than other patients would.

Some studies suggested that median survival times were longer among patients expressing low levels of CALCRL, both in patients without t(8 ; 21) (Supplementary Figure S2) [19] and in AML-M2 patients (Supplementary Figures 3A). The *CALCRL* played an important role in stemness and chemotherapy resistance in AML. Knockdown of *CALCRL* expressed in the leukemic stem cell (LSC) subpopulation decreased the LSC frequency and sensitized it to chemotherapeutic agents, facilitating to eradicate the relapse-initiated cells (RICs) in AML [8, 9, 20, 21]. In patients with t(8 ; 21), there was only a trend shown but did not reach statistical difference due to the low number of samples (Supplementary Fig. S3BC) [22]. Consistent with these findings, our results suggest that *CALCRL* expression may be related to tumor burden and prognosis in AML/ETO^+^ AML patients as well. Importantly, antibodies interfering with CALCRL signaling have recently been approved for the preventive treatment of migraine headaches [23–25]. All of these results indicate that drugs targeting CALCRL may be suitable add-ons for AML therapy in the context of AML.

## 5. Conclusions

Our study indicates that CALCRL could serve as a suitable prognostic factor for designing the chemotherapy regimen and evaluating the risk of HSCT in AML/ETO^+^ AML patients. Our experimental results need to be confirmed with larger patient cohorts.

## Figures and Tables

**Table 1:** Relationship between the expression levels of *CALCRL* mRNA and basic clinical features.

Clinical features	CALCRL^low^ (*n* = 34)	CALCRL^high^ (*n* = 33)	*P* value
Gender (male/female)	18/16	22/11	0.3702
Age (year)	36 (15–58)	35 (14–57)	0.8951
WBC × 1012^/^L	14.04 (0.8–79.05)	21.55 (2.64–200)	0.2582
N × 109/L	74.24 (35–115)	67.91 (47–135)	0.2044
PLT × 109/L	28.53 (6–93)	27.12 (8–89)	0.7728
HGB g/L	46.16 (18–80.5)	51.65 (13–87)	0.2575
Blast in BM (%)	46.00 (0.4–88)	50.74 (6.4–88.1)	0.5086
Hazard stratification			0.936
Low risk	10	11	
Medium risk	16	15	
High risk	8	7	

**Table 2:** Relationship between *CALCRL* mRNA expression levels and gene mutations.

Clinical features	CALCRL^low^ (*n* = 34)	CALCRL^high^ (*n* = 33)	*P* value
All mutations	27	29	
c-Kit	14	25	0.0082
*CEBPA*	2	0	0.2279
*FLT3*	6	2	0.137

## Data Availability

Data and materials would be made available upon request.

## Abbreviations

CALCRL: Calcitonin receptor-like gene
AML: Acute myeloid leukemia
MICM: Morphology, immunology, cytogenetic, and molecular biology
MNCs: Marrow mononuclear cells
CR: Clinical remission
HSCT: Hematopoietic stem cell transplantation.

## Supplementary Materials

Supplementary Materials Supplementary Figure S1: CALCRL was related to the OS in AML. Microarray analysis identified 1613 differentially expressed genes that had <0.5-fold changes and *p* < 0.05 after H22954 overexpression (left circle). BLAST analysis shown that 400 human genes were identified as potential H22954 target genes in the TargetScanHuman 6.0 database (right circle); 81 genes common in both sets are shown in the box. CALCRL was one of these 81 genes which mostly related to the OS in AML. Supplementary Figure S2: CALCRL was related to the OS in AML patients without t(8 ; 21) in GSE14468 datasets. Supplementary FigS3: A. CALCRL was related to the OS in AML-M2 in TCGA datasets. B. CALCRL was related to the OS in AML patients with t(8 ; 21) in TCGA datasets. C. CALCRL was related to the OS in AML patients with t(8 ; 21) in TARGE datasets.
