# Supplementary figures and images for: CALCRL Gene is a Suitable Prognostic Factor in AML/ETO+ AML Patients
Source: J Oncol. 2022 Mar 16;2022:3024360. doi: 10.1155/2022/3024360 (PMC8942673; doi:10.1155/2022/3024360)

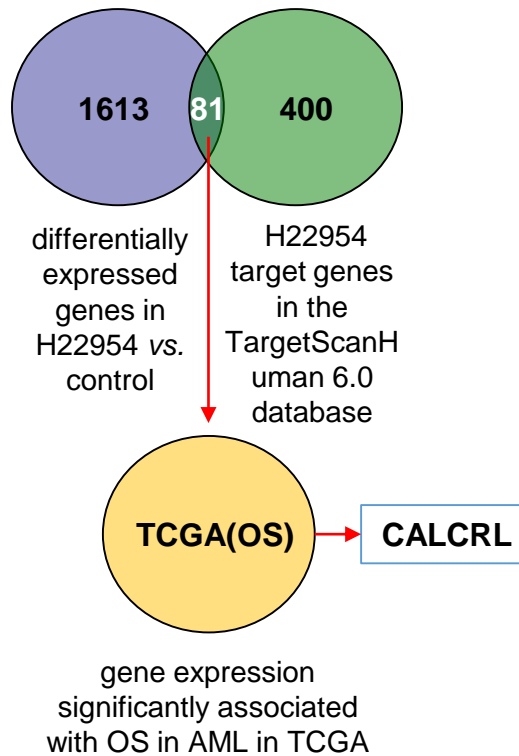

Supplement Figure S1

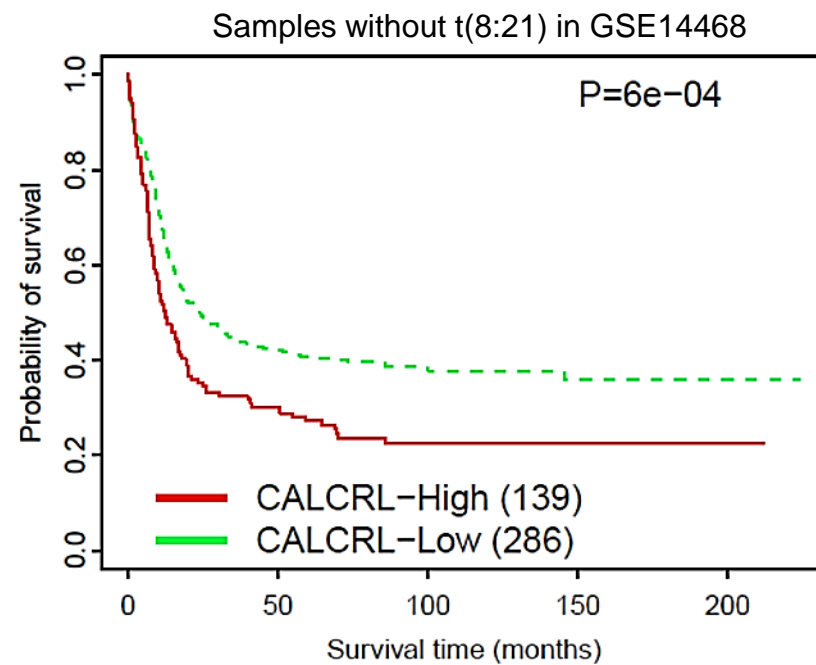

A

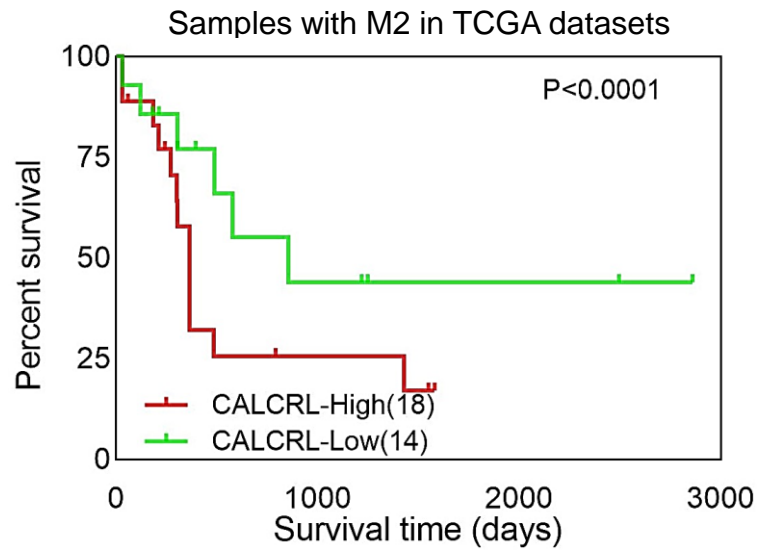

B

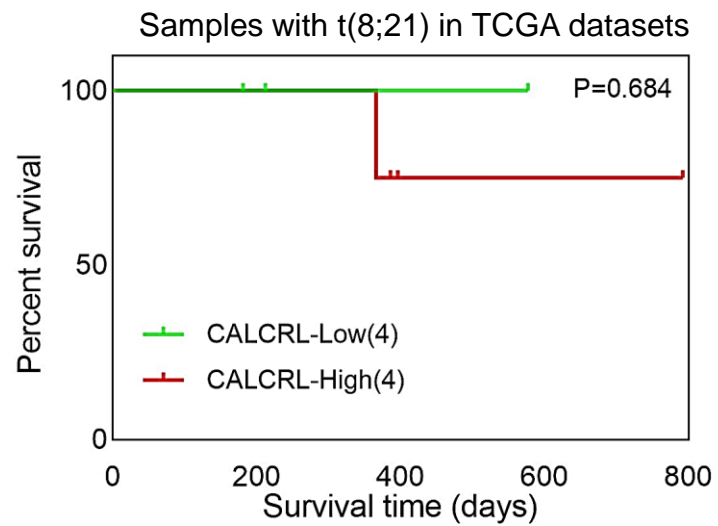

C

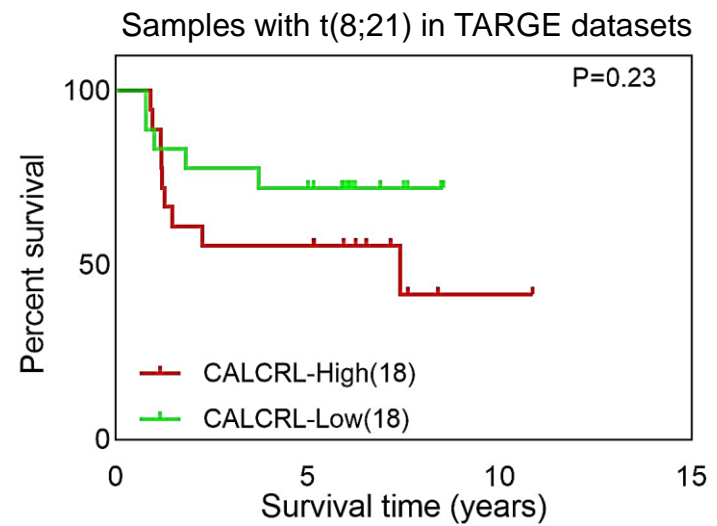

Supplement: Supplementary Materials — Supplementary Figure S1: CALCRL was related to the OS in AML. Microarray analysis identified 1613 differentially expressed genes that had <0.5-fold changes and p < 0.05 after H22954 overexpression (left circle). BLAST analysis shown that 400 human genes were identified as potential H22954 target genes in the TargetScanHuman 6.0 database (right circle); 81 genes common in both sets are shown in the box. CALCRL was one of these 81 genes which mostly related to the OS in AML. Supplementary Figure S2: CALCRL was related to the OS in AML patients without t(8 ; 21) in GSE14468 datasets. Supplementary FigS3: A. CALCRL was related to the OS in AML-M2 in TCGA datasets. B. CALCRL was related to the OS in AML patients with t(8 ; 21) in TCGA datasets. C. CALCRL was related to the OS in AML patients with t(8 ; 21) in TARGE datasets. [file 3024360.f1.pdf]
